# Chloride Diffusion in Concrete Protected with a Silane-Based Corrosion Inhibitor

**DOI:** 10.3390/ma13082001

**Published:** 2020-04-24

**Authors:** Luigi Coppola, Denny Coffetti, Elena Crotti, Gabriele Gazzaniga, Tommaso Pastore

**Affiliations:** 1 Department of Engineering and Applied Sciences, University of Bergamo, 24044 Dalmine (BG), Italy; denny.coffetti@unibg.it (D.C.); elena.crotti@unibg.it (E.C.); gabriele.gazzaniga@unibg.it (G.G.); tommaso.pastore@unibg.it (T.P.); 2 UdR Materials and Corrosion, Consorzio INSTM, 50121 Florence, Italy; 3 UdR Bergamo, Consorzio CSGI, 50019 Sesto Fiorentino, Italy

**Keywords:** durability of concrete, chloride penetration, rebar corrosion, corrosion inhibitor, silane-based surface treatment

## Abstract

One of the most important parameters concerning durability is undoubtedly represented by cement matrix resistance to chloride diffusion in environments where reinforced concrete structures are exposed to the corrosion risk induced by marine environment or de-icing salts. This paper deals with protection from chloride ingress by a silane-based surface-applied corrosion inhibitor. Results indicated that the corrosion inhibitor (CI) allows to reduce the penetration of chloride significantly compared to untreated specimens, independently of w/c, cement type, and dosage. Reduction of chloride diffusion coefficient (D_nssn_) measured by an accelerated test in treated concrete was in the range 30–60%. Natural chloride diffusion test values indicate a sharp decrease in apparent diffusion coefficient (D_app_) equal to about 75% when concrete is protected by CI. Mechanism of action of CI in slowing down the chloride penetration inside the cement matrix is basically due to the water repellent effect as confirmed by data of concrete bulk electrical resistivity.

## 1. Introduction

Concrete alkalinity promotes the formation of a passive protective oxide layer able to prevent corrosion of steel rebars and guarantees an adequate service life of reinforced concrete structures [1]. However, de-passivation of reinforcements can take place for many reasons, among which the most widespread is when chlorides reach a critical concentration at the interface cement matrix/steel bar. Chlorides can penetrate inside the cement matrix from external sources by capillary suction or by diffusion, for example, from contact or proximity to sea water or in a structure where de-icing salts are used, but can also be added incorrectly into the concrete through contaminated aggregates, admixtures, or water [2,3]. It is well known that the chloride-induced corrosion is one of the most dangerous and common phenomena for reinforced concrete structures in the marine environment or exposed to de-icing salts [4]. In a perspective of sustainability in the construction sector and to prevent premature structural failures due to chloride-induced corrosion, it is important to investigate possible strategies to counteract this degradation phenomenon [5,6]. Before dealing with these preventive methods, it is important to underline how the correct choice of concrete cover and mixture composition plays an important role in hindering the diffusion process of chlorides inside the cement matrix [7,8]. In agreement with the diagram of Tuuti [9], one of the main goals consists in slowing down the chloride diffusion inside the matrix in order to delay the onset of the corrosion process. Several alternative strategies have been proposed for increasing the durability of reinforced concrete structures exposed to chloride-rich environments such as coatings [10,11], cathodic protection [12,13], chloride extraction [14], and use of corrosion inhibitors [15,16,17,18,19]. Among these, the use of corrosion inhibitors (CI) is one of the most effective and cheaper ways to prevent the chloride-induced corrosion of reinforced concrete structures. 

Two different types of corrosion inhibitors are available on the market: the admixed inhibitors, added to fresh concrete, and migrating corrosion inhibitors—also called penetrating inhibitors or surface-applied corrosion inhibitors—applied on the hardened concrete surface [20]. In particular, the latter seems to be an interesting solution for existing concrete structures exposed to chlorides such as infrastructures, bridges, marine structures, seawater pipelines, and chemical industries [21]. Many investigations have been conducted on surface-applied corrosion inhibitors. Soylev et al. evidenced the effectiveness of amino alcohol-based surface-applied corrosion inhibitors due to a pore-blocking effect as demonstrated by the resistivity measurements of concrete [22]. However, the inhibitors seem to block the pores on the surface of concrete rather than the bulk concrete similarly to a waterproofing treatment [23]. Holloway et al. found that the corrosion inhibitor was still present in the concrete cover at 5 years from application [24]. Research by Fedrizzi et al. demonstrated that the simultaneous use of the alkanolamine-based inhibitor with a good barrier coating offers protection against chloride-induced rebar corrosion [25]. Finally, the efficiency of a surface-applied corrosion inhibitor based on alkylaminoalcohol was highlighted by Morris and Vazquez, especially when it was applied on low-quality concretes manufactured with raw materials contaminated with chloride ions [26]. 

The purpose of this paper is to evaluate the performances of a silane-based corrosion inhibitor applied on the surface of concrete element in order to slow down chloride diffusion in cement matrix and, consequently, to delay the onset of the corrosion process. The experimental program was carried out both in the form of accelerated and natural diffusion tests in different concrete mixtures manufactured in order to evaluate—other than the efficiency of CI treatment—the influence of w/c, cement type, and cement factor on the penetration mechanism. For each concrete, the chloride diffusion coefficient (D_nssm_ also called D_RCM_) was calculated to quantify the reduction in the penetration of chloride into the cement matrix in accordance with Spiesz and Brouwers [27] and Li et al. [28].

## 2. Materials and Methods 

**Figure 1:**
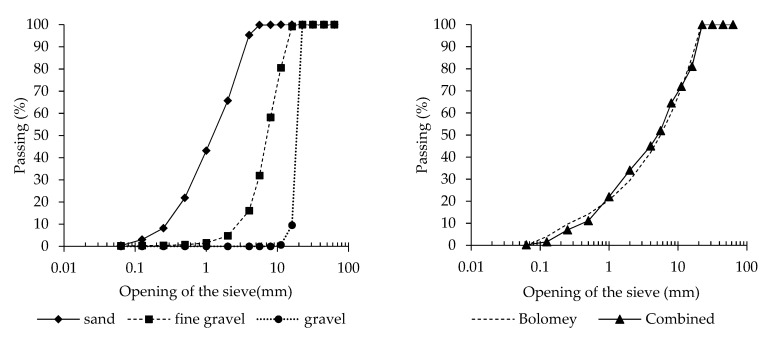
Grading curves of the aggregates (**left**). Bolomey and combined aggregate curves (**right**).

Seven different types of concrete have been manufactured (Table 1). Water/cement ratio and cement factor were selected in order to meet requirements for the exposure classes XD and XS according to EN 206 [29]. The denomination of the different concretes was made taking into consideration the different variables analysed: type of cement (natural pozzolanic cement: CEM IV/A-P 42.5R, limestone Portland cement: CEM II/A-L 42.5R, and blast furnace cement: CEM III/A 42.5R—Table 2), w/c ratio (0.55, 0.50, and 0.45) and cement factor (320, 340, and 360 kg/m^3^). Finally, natural siliceous sand and gravel (three different gradings) with a maximum size equal to 22 mm were combined in order to meet the Bolomey curve (Figure 1).

At the end of the mixing procedure, workability was measured by flow table according to EN 12350-5 [30]. In addition, specific mass and entrapped air were evaluated on fresh concretes according to EN 12350-6 [31] and EN 12350-7 [32] standards, respectively. For each concrete mixture, 30 cubic specimens (150 × 150 mm) and 32 cylindric specimens (d = 100 mm and h = 200 mm) were manufactured. Concrete samples were removed from the steel molds after 24 h and subsequently cured according to the scheme in Table 3. Compressive strength on hardened concrete was also determined at different ages (EN 12390-3 [33]). 

For the estimation of chloride penetration into concrete, accelerated migration tests, natural diffusion tests, and bulk electrical resistivity measurements were carried out on concrete with and without silane-based surface treatment as detailed in the following paragraphs. In particular, after a proper curing time (Table 3), half of the specimens were subjected to a silane-based surface-applied corrosion inhibitor while the others were used as an untreated reference. The properties of the corrosion inhibitor and application procedure are reported in Table 4.

### 2.1. Accelerated Chloride Migration Tests

Accelerated chloride migration tests were carried out according to NT BUILD 492 [34]. A cylindrical water-saturated concrete specimen (100 mm diameter and 50 mm height) was placed between two cells, one of them filled with 0.30 N NaOH solution and the other with a 10 wt.% NaCl solution. A 30 V DC potential was applied across the sample and the initial current was evaluated. Based on the measured initial current, the test voltage and the test duration were selected according to the NT BUILD 492. A data logger (Germann Instruments Ltd., Copenhaghen, Denmark) was used to record the electrical current, the temperature, and the electrical permeability during the test. Finally, the penetration depth of chlorides was determined by means of a 0.1 M silver nitrate solution [35,36] and the chloride diffusion coefficient (D_nssm_) was calculated by the following equation [27]:

(1) $$D_{\mathrm{nssm}}=\frac{\mathrm{RT}}{\mathrm{zFE}}\;\times \;\frac{x_{d}\;-\;\alpha \sqrt{x_{d}}}{t}$$

with

(2) $$E=\frac{U\;-\;2}{L},$$

(3) $$\alpha =2\sqrt{\frac{\mathrm{RT}}{\mathrm{zFE}}}{\mathrm{erf}}^{-1}\left(1\;-\;\frac{{2c}_{d}}{c_{0}}\right)$$

where z is the absolute value of ion valence, F is the Faraday constant, U is the absolute value of the applied voltage, R is the gas constant, T is the average value of the initial and final temperatures in the anolyte solution, L is the thickness of the specimen, X_d_ is the average value of the penetration depths, t is the test duration, erf^−1^ is the inverse of error function, C_d_ is the chloride concentration at which the color changes, and C_0_ is the chloride concentration in the catholyte solution. 

### 2.2. Natural Chloride Diffusion Tests

For the natural chloride diffusion test, 150 mm cubic specimens were stored in a 3 wt.% NaCl solution at 20 °C for six months. The solution was replaced monthly and, at fixed intervals (1–2–3–6 months), the samples were split into two halves by means of a compression testing machine (Controls Spa, Liscate (MI), Italy) and the penetration of chlorides was measured using the previously described colorimetric method based on silver nitrate [35,36].

### 2.3. Bulk Electrical Resistivity Tests

The standard method reported in ASTM C1760 was used to evaluate the bulk electrical resistivity of concrete with and without corrosion inhibitor. The water-saturated concrete sample (100 mm diameter and 100 mm height) was positioned between the test cells used for accelerated chloride migration test containing 3 wt.% NaCl solution and an electrical potential of 60 V DC was applied across the specimen. The bulk electrical resistivity was calculated using the following equation:

(4) $$\rho =\frac{V}{I}\times \frac{\pi d^{2}}{4L}$$

where ρ is the electrical resistivity in kΩ·cm, V is the applied voltage (60 V), I is the current in A, d is the specimen diameter (100 mm), and L is the specimen length (100 mm).

## 3. Results and Discussion

### 3.1. Fresh Properties

In Table 5, the fresh properties of concretes are listed. No substantial differences between the different mixtures in terms of workability, air content, and specific mass at fresh state were noticed. All concretes evidenced workability class F4 according to EN 206 [29] and the air content reflects the one expected for concrete manufactured with aggregate having maximum size equal to 22 mm. Finally, the specific mass at fresh state is similar for all concretes investigated.

### 3.2. Elasto-Mechanical Properties

Table 6 shows results of compressive strength at different ages; as expected, the lower the w/c, the higher the compressive strength values.

Compressive strength at 210 days is 26–30% higher than the corresponding value achieved at 28 days for concretes manufactured with pozzolanic (IV) and blastfurnace (III) cements. The 210-day strength value of limestone Portland cement concrete (II), on the contrary, is only 18% higher than the 28-day compressive strength. Data confirm that when a pozzolanic or blastfurnace cement is used, a higher increase of compressive strength with time is achieved as a consequence of the pozzolanic reaction [37].

### 3.3. Bulk Electrical Resistivity Tests Results

The average bulk electrical resistivity of water-saturated concretes is reported in Table 7. As shown, all values related to untreated concretes are in the range of 5–8 kΩ·cm after 7 days and 8–14 kΩ·cm after 28 days, in accordance with Layssi et al. [38] and Neville [37]. Small differences are detected by varying the cement factor at equal w/c; on the contrary, the electrical resistivity increase when low w/c was adopted and it decreases when limestone Portland cement (II) was used instead of pozzolanic cement (IV) or blastfurnace (III) cement due to the denser structure promoted by the pozzolanic reaction of slag and natural pozzolan [39]. The use of a surface-applied corrosion inhibitor on concrete determines a strong increase in electrical resistivity, both at 7 and 28 days. However, the increasing in electrical resistivity is higher at 7 days (about +85%–+145%) respect to that at 28 days (about +40%–+65%).

**Figure 2:**
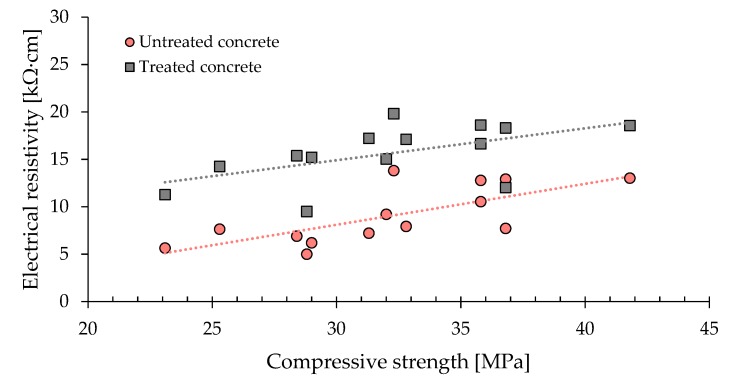
Correlation between electrical resistivity and compressive strength for treated and untreated concrete.

**Figure 3:**
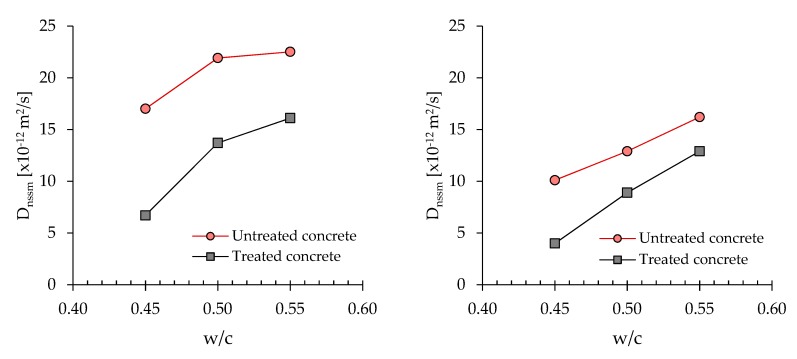
Chloride diffusion coefficient (D_nssn_) vs. w/c ratio at 7 days (**left**) and 28 days (**right**).

Figure 2 shows the bulk electrical resistivity of both treated and untreated specimens as a function of compressive strength of concrete. Figure 3 clearly confirms a significant increase in the electrical resistivity as a consequence of the surface treatment by CI and it seems to indicate that the surface-applied corrosion inhibitors acts as a water repellent protection. 

Data highlight the positive role of w/c since the electrical resistivity increases with concrete compressive strength independently of whether the specimen is treated or not, confirming results available in literature [40,41,42]. Moreover, the slope of the trend line for treated specimens is higher than that of untreated concrete. Assuming the strong direct relationship between the electrical resistivity and chloride diffusion reported in several papers [43,44,45], data reported in Figure 3 indicate that the corrosion inhibitor is more effective in slowing down chloride diffusion in concretes having high mechanical performances.

### 3.4. Accelerated Chloride Migration Tests Results

The chloride diffusion coefficient (D_nssn_) resulting from the accelerated diffusion test basically depends on the depth of chloride penetration in concrete (Table 8). As expected, the concretes manufactured with pozzolanic cement (IV) or blastfurnace cement (III) show a lower chloride diffusion coefficient with respect to limestone Portland cement-based mixtures (II). In particular, the D_nssm_ is in the range of 14–22 × 10^−12^ m^2^/s at 7 days and 6–16 × 10^−12^ m^2^/s at 28 days for III and IV samples, while II specimens reach values close to 28 × 10^−12^ m^2^/s and 21 × 10^−12^ m^2^/s, respectively. Protecting the concrete surface by the CI treatment determines a significant reduction of chloride penetration, independently of the age of concrete (7 or 28 days) when the accelerated diffusion test is carried out. The reduction of D_nssm_ is close to 30–40% if measured on samples water cured for 7 days and it slightly decreases at 21–39% when concrete is cured 28 days. 

Figure 3 reports the chloride diffusion coefficient (D_nssn_) of pozzolanic cement-based concretes (IV) as a function of w/c ratio. Data are in good agreement with electrical resistivity results confirming the effectiveness of CI treatment in preventing chloride ingress inside the matrix. Moreover, according to electrical resistivity data, the efficiency of the CI treatment seems to be higher than the lower the w/c. 

**Figure 4:**
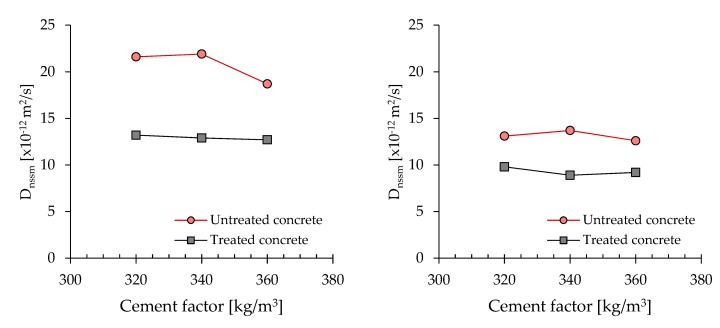
Chloride diffusion coefficient (D_nssn_) vs. cement dosage at 7 days (**left**) and 28 days (**right**).

Figure 4 presents the chloride diffusion coefficient (D_nssn_) vs. cement factor for concrete manufactured with CEM IV/A-P 42.5 R at the same w/c (0.50). Results confirm the positive role of the CI treatment, independently of the cement dosage. Similar to the bulk electrical resistivity, the chloride diffusion coefficient is not strongly influenced by the cement factor. Experimental results are in agreement with Bertolini et al. [46], affirming the binder content of the cement-based mixtures does not entail significant differences in terms of resistance to the penetration of chlorides.

**Figure 5:**
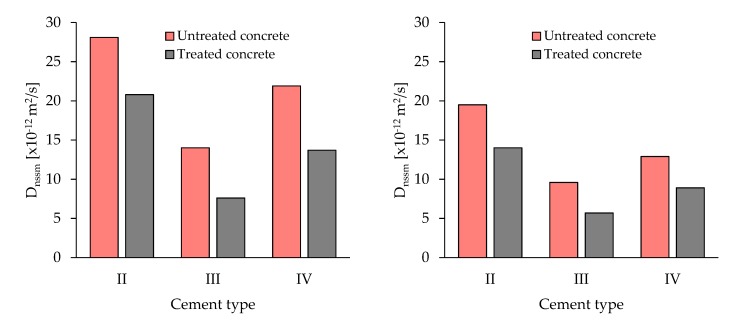
Chloride diffusion coefficient (D_nssn_) vs. cement type for concrete manufactured with the same w/c (0.50) at 7 days (**left**) and 28 days (**right**).

Figure 5 shows chloride diffusion coefficient (D_nssn_) vs. cement type for concrete manufactured with the same w/c (0.50). Data confirm the efficiency of CI treatment independently of the cement type. Results also indicate the positive role of pozzolanic and blastfurnace cement in reducing the chloride penetration inside the matrix as a consequence of the binding capacity of pozzolanic reaction products [47,48,49].

On the basis of the experimental results of the accelerated chloride diffusion test, it is possible to affirm that the surface-applied corrosion inhibitor performs better if applied on concrete intrinsically resistant to chloride penetration manufactured with a pozzolanic or blastfurnace cement and with a low w/c ratio.

**Figure 6:**
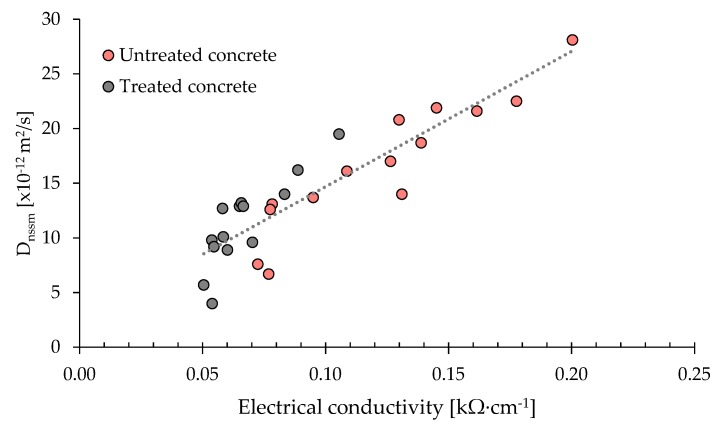
Correlation between chloride diffusion coefficient and electrical conductivity of concrete.

Finally, from the analysis of parameters resulting from the accelerated chloride migration tests and the bulk electrical resistivity tests, it was possible, in accordance with Layssi et al. [38], to correlate the chloride diffusion coefficient and the electrical conductivity of concrete (Figure 6). A linear correlation can be found, in accordance with the Nernst–Einstein equation, that can lead to hypothesize that the protective corrosion inhibitor acts only in terms of increasing electrical resistivity (water repellent effect) without chemically modifying the ability to bind chloride ions.

### 3.5. Natural Migration Test

**Figure 7:**
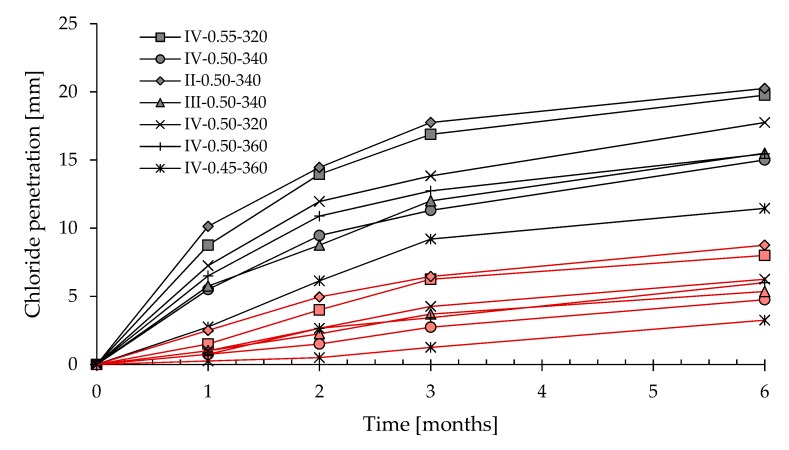
Correlation between chloride penetration and time (untreated concrete in black, treated concretes in red).

Figure 7 shows chloride penetration vs. time for concrete specimens immersed in 3 wt.% NaCl aqueous solution. After 6 month of immersion, untreated samples evidenced a chloride penetration in the range of 11–20 mm, while the penetration depth of treated specimens is about 3–8 mm. Results clearly indicate that the CI treatment is strongly efficient in reducing the chloride diffusion independently of the w/c, the type and the dosage of cement, confirming the results registered for the accelerated chloride diffusion test.

**Figure 8:**
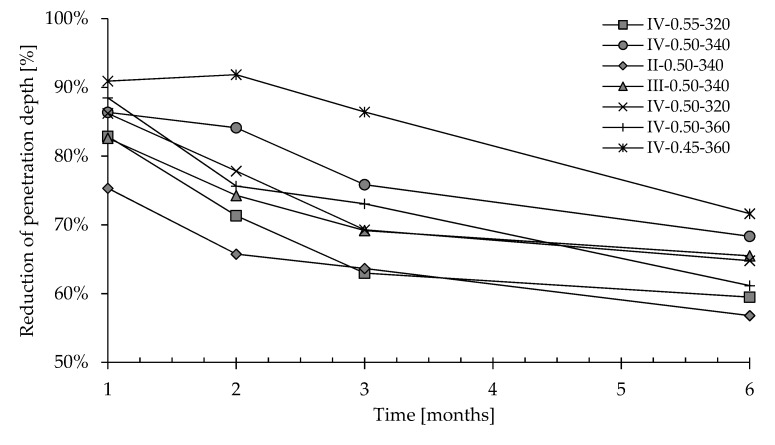
Reduction of depth penetration after natural migration test for different concretes.

After three months of exposure, a reduction of 65–90% could be noticed in chloride penetration as a consequence of the CI treatment (Figure 8). After 6 months, concrete depth penetrated by chloride in treated samples is lower than that measured in untreated specimens of about 55–75%. Data seem to indicate that the efficiency of CI treatment in slowing down chloride diffusion slightly decreases with time. This behavior could be attributable to a partial leaching of the corrosion inhibitor as a consequence of the permanent immersion in chloride-based solution as already hypothesized by Zheng et al. [50]. Further results at ages longer than 6 months are in progress to understand if efficiency of the surface-applied corrosion inhibitor remains constant or decreases with time.

Chloride penetration values (X) measured from the natural diffusion test over six months are used for the calculation of the average value (Table 9) of apparent diffusion coefficient (D_app_) according to the following equation [51]:

(5) $$0.66X=1206\sqrt{9.46\times 10^{7}\times T_{\mathrm{SLS}}\times D_{\mathrm{app}}}+\mathrm{dx}$$

where 0.66 X coincides with the depth at which the critical concentration of chlorides is reached (0.4% respect to cement mass), T_SLS_ is the duration of the exposure to the chloride-rich solution expressed in years, and dx is the thickness of the convection layer depending on the concrete compressive strength. At equal w/c (0.50), D_app_ values are in the range of 0.20–0.30 × 10^−12^ m^2^/s for untreated concretes manufactured with blastfurnace (III) or pozzolanic (IV) cements while limestone Portland cement-based mixtures (II) evidenced higher apparent diffusion coefficients, close to 1.15 × 10^−12^ m^2^/s. The reduction in w/c promotes the formation of denser cementitious matrix with low D_app_ in accordance with the study of Neville [37].

Treatment by the corrosion inhibitor determines a sharp decrease of D_app_; values of treated concrete are in the range of 0.03–0.21 × 10^−12^ m^2^/s, about 75% lower than those detected for concretes without treatment. The efficiency of CI treatment seems to be independent of w/c, type, and dosage of cement. However, the lowest values for D_app_ were obtained for those concretes intrinsically resistant to chloride penetration (low w/c and pozzolanic or blastfurnace cement), confirming the same results obtained for the accelerated chloride diffusion test.

**Figure 9:**
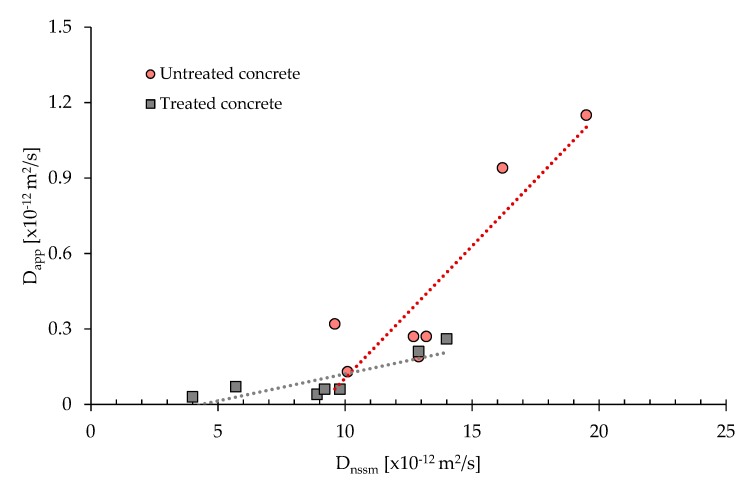
Correlation between D_nssm_ and D_app_.

Figure 9 compares D_app_ and 28-day D_nssn_; the correspondence between the two coefficients is linear and the proportionality factor is consistent, as reported in study of Spiesz and Brouwers [27]. In particular, the regression line of treated specimens is placed below that of untreated concretes and the slope is lower compared to the same value of untreated samples. These two aspects confirm that the CI protective treatment is particularly effective since the increase of the D_nssm_ determines a slower growth of D_app_ in treated specimens.

## 4. Conclusions

The following conclusions could be drawn from the present study:

- The surface-applied corrosion inhibitor allows to reduce significantly the penetration of chloride in concrete, independently of w/c, cement type, and dosage. Reduction of the chloride diffusion coefficient (D_nssn_) measured by an accelerated test in treated concrete was in the range 30–60%. Natural chloride diffusion test values indicate a sharp decrease in D_app_ equal to about 75% when concrete is protected by the surface-applied CI.
- Mechanism of action of CI in slowing down the chloride penetration inside the cement matrix is basically due to the water repellent effect as confirmed by data of concrete electrical resistivity and accelerated chloride migration test results.
- The w/c ratio has confirmed to have a significant influence on chloride diffusion: the lower the w/c, the lower the penetration inside the cement matrix. On the contrary, no significant differences are observed in chloride penetration changing the cement dosage at the same w/c ratio.
- The type of cement considerably affects the chloride diffusion in concrete; in particular, it has been confirmed that limestone Portland cement (II) should be avoided in environments rich in chlorides, preferring pozzolanic (IV) or blast furnace (III) cements.

Further experimental data are in progress to evaluate the effectiveness of the surface-applied corrosion inhibitor at very long ages. Moreover, further studies should be focused on understanding if the migrating corrosion inhibitor is capable to stop corrosion in chloride contaminated concrete where the critical chloride concentration is reached on the steel rebars.

## Figures and Tables

**Table 1:** Composition of the concretes.

Concrete	CEM IV/A-P 42.5 R [kg/m^3^]	CEM II/A-LL 42.5 R [kg/m^3^]	CEM III/A 42.5 R [kg/m^3^]	Aggregates [kg/m^3^]	Water [kg/m^3^]	w/c
IV-0.55-320	320			1880	176	0.55
IV-0.50-340	340			1875	170	0.50
II-0.50-340		340		1885	170	0.50
III-0.50-340			340	1875	170	0.50
IV-0.50-320	320			1915	160	0.50
IV-0.50-360	360			1830	180	0.50
IV-0.45-360	360			1885	162	0.45

**Table 2:** Main properties of cements.

Properties	CEM IV/A-P 42.5 R	CEM II/A-LL 42.5 R	CEM III/A 42.5 R
Specific mass [kg/dm^3^]	3.01	3.10	3.05
Specific surface [m^2^/kg]	480	400	400
Setting time [min]	>130	>130	>60

**Table 3:** Specimens, curing procedure, and preparation.

Test	Curing and Preparation	Specimen Format	Note
Compressive strength	Curing at 20 °C and R.H. > 95% until the deadline	Cube 150 mm	1–7–28–70–100–130–210 days; 2 samples for each age
Accelerated chloride diffusion test - Bulk electrical resistivity test	Curing at 20 °C and R.H. > 95% for 7 days; Preparation of specimens by sawing and grinding; Drying in oven at 60 °C; Application of the CI; Water saturation for 24 h of samples for 7-day tests; Soaking the specimen in water for 28-day tests	Cylinder d: 100 mm h: 50 mm - d: 100 mm h: 100 mm	7–28 days; 8 samples for each age (4 treated and 4 untreated)
Natural chloride diffusion test	Curing at 20 °C and R.H. > 95% for 14 days; Curing at 20 °C and R.H. 60% for 28 days; Application of the CI; Immersion of specimens in a 3 wt.% NaCl solution until the deadline	Cube 150 mm	1–2–3–6 months of immersion; 4 samples for each age (2 treated and 2 untreated)

**Table 4:** Properties of the corrosion inhibitor and application procedure.

Properties	Value
Color	Straw yellow
Viscosity [mPa·s]	0.95 ± 0.05
Dry residue [%]	7 ± 0.3
pH	6.5 ± 0.2
Density [kg/dm^3^]	0.88 ± 0.05
Average consumption [L/m^2^]	0.25 for each coat
Number of coats	4
Time between coats	15 min
Application method	Brush

**Table 5:** Properties of concretes at fresh state.

Concrete	Workability [mm]	Air Content [%]	Specific Mass [kg/m^3^]
IV-0.55-320	550	1.6	2375
IV-0.50-340	520	1.7	2380
II-0.50-340	530	1.8	2395
III-0.50-340	540	1.9	2385
IV-0.50-320	530	1.9	2395
IV-0.50-360	530	1.8	2375
IV-0.45-360	510	1.6	2405

**Table 6:** Cubic compressive strength (fc) results.

Concrete	w/c Ratio	Cubic Compressive Strength: fc [MPa]							
		1 d	7 d	28 d	70 d	100 d	130 d	210 d	210 d-fc/28 d-fc
IV-0.55-320	0.55	11.1	23.1	32.0	36.6	39.4	39.9	41.2	129%
IV-0.50-340	0.50	13.5	28.4	35.8	40.8	42.5	43.5	46.4	130%
II-0.50-340	0.50	16.7	28.8	36.8	39.8	41.4	43.1	43.4	118%
III-0.50-340	0.50	13.5	25.3	32.3	38.1	40.9	41.9	42.9	133%
IV-0.50-320	0.50	13.8	29.0	35.8	39.7	43.0	43.3	45.0	126%
IV-0.50-360	0.50	17.5	31.3	36.8	40.5	43.7	45.0	46.9	127%
IV-0.45-360	0.45	17.8	32.8	41.8	45.8	48.8	50.0	53.1	127%

**Table 7:** Bulk electrical resistivity tests results.

Concrete	Bulk Electrical Resistivity at 7 d [kΩ·cm]		Bulk Electrical Resistivity at 28 d [kΩ·cm]	
	Untreated	Treated	Untreated	Treated
IV-0.55-320	5.6	11.3	9.2	15.0
IV-0.50-340	6.9	15.4	10.5	16.6
II-0.50-340	5.0	9.5	7.7	12.0
III-0.50-340	7.6	13.3	13.8	19.8
IV-0.50-320	6.2	15.2	12.8	18.6
IV-0.50-360	7.2	17.2	12.9	18.3
IV-0.45-360	7.9	17.1	12.0	18.5

**Table 8:** Values of chloride diffusion coefficient of concretes.

Concrete	D_nssm_ [×10^−12^ m^2^/s]					
	Untreated Specimens		Treated Specimens		Reduction [%]	
	7 d	28 d	7 d	28 d	7 d	28 d
IV-0.55-320	22.5	16.1	16.4	12.9	27.1	21.3
IV-0.50-340	21.9	13.7	12.9	8.9	41.1	35.1
II-0.50-340	28.1	20.8	19.5	14.0	30.8	32.7
III-0.50-340	14.0	7.6	9.6	5.7	31.4	25.0
IV-0.50-320	21.6	13.1	13.2	9.8	38.9	25.2
IV-0.50-360	18.7	12.6	12.7	9.2	32.1	27.0
IV-0.45-360	17.0	6.7	10.1	4.0	40.6	39.4

**Table 9:** Average values of D_app_ for different concretes.

Concrete	D_app_ [×10^−12^ m^2^/s]		Reduction [%]
	Untreated Specimens	Treated Specimens	
IV-0.55-320	0.94	0.21	77.6
IV-0.50-340	0.19	0.04	78.9
II-0.50-340	1.15	0.26	77.4
III-0.50-340	0.32	0.07	78.1
IV-0.50-320	0.27	0.06	77.8
IV-0.50-360	0.24	0.06	75.0
IV-0.45-360	0.13	0.03	76.9
